# Self-assessed quality of care among adults with diagnosed diabetes in Germany

**DOI:** 10.25646/8329

**Published:** 2021-06-16

**Authors:** Jens Baumert, Rebecca Paprott, Yong Du, Christin Heidemann, Christa Scheidt-Nave

**Affiliations:** Robert Koch Institute, Berlin, Department of Epidemiology and Health Monitoring

**Keywords:** DIABETES, QUALITY OF CARE, SUBJECTIVE ASSESSMENT, POPULATION, DIABETES SURVEILLANCE

## Abstract

People who have diabetes require regular medical care. The views of patients about the quality of their care are becoming increasingly relevant when it comes to chronic diseases such as diabetes. As part of the nationwide study Disease Knowledge and Information Needs - Diabetes mellitus (2017), data on self-assessed quality of care by people with diagnosed diabetes was collected using the Patient Assessment of Chronic Illness Care - DAWN short form (PACIC-DSF, scale 1 to 5) and analysed for respondents aged 45 years or above. The average score for quality of care was 2.47 and was lower for women than for men (2.33 vs 2.58). The respondents assessed the quality of their care as being worse with rising age and size of the population in their residential area. No significant differences were observed by education group. Overall, people with diabetes in Germany consider the quality of their care to be moderate, which indicates a need for improvement in care.

## Introduction

Diabetes mellitus is a metabolic disease resulting from disorders in the regulation of blood sugar levels [1] and belongs to the chronic diseases with a high frequency (prevalence) [2]. Diabetes is associated with an increased risk of serious comorbidities and secondary diseases [3] as well as increased mortality [4]. Consequently, people with diabetes need regular, well-coordinated medical care in addition to good self-management [5].

These needs have led to the establishment of evidence-based national guidelines and structured care programmes (disease management programmes) in various countries, including Germany, to ensure that people with diabetes receive a high quality of care [6, 7]. These guidelines and programmes are widely used in practice and include recommendations on medicinal treatment (such as when insulin is necessary), therapy goals (such as controlling blood sugar levels and additional cardiovascular risk factors), self-management (such as self-monitoring of blood sugar levels) and follow-up checks for the early detection of diabetes-related complications. Since its establishment at the Robert Koch Institute (RKI) in 2015, the diabetes surveillance system has been using selected core indicators denoting the quality of care [8] to analyse both the extent to which guideline-based recommendations on the quality of care for people with diabetes are being implemented in Germany, and whether it is changing over time. To this end, data collected from the ongoing DMP documentation compiled by the Central Research Institute of Ambulatory Health Care in Germany (Zi) [9] and from nationwide health surveys are used [10].

In addition to this objective assessment of the quality of care using data on the implementation of the guidelines, the subjective assessment of the quality of care from the perspective of the people affected by diabetes is becoming increasingly important [11]. Self-assessed quality of care is one of the ten supplementary indicators comprising the indicator set of the diabetes surveillance in Germany. Epidemiological studies on self-assessed quality of care in adults with diabetes have not been available for Germany to date. The aim of this study, therefore, is to examine how people with diabetes in Germany assess the quality of the care they receive.

## Indicator

**Info box Studying self-assessed quality of care using the Patient Assessment of Chronic Illness Care – DAWN short form**

**Introductory question:**

What kind of help have you received from your health care team for diabetes management in the past twelve months?

**Individual questions:**

1. I was asked how diabetes affects my life.
2. I was asked about the effectiveness of my medication and any problems and side effects that may have occurred.
3. I was asked for my wishes and goals when the treatment plan for my diabetes was being drawn up.
4. I was supported in setting specific goals to improve my diabetes management.
5. I was supported in developing plans to meet my diabetes treatment goals.
6. I was supported in developing plans for how I could get support from friends, family or people around me.
7. I was encouraged to attend a specific group or class that will help me manage my diabetes.
8. I was contacted after my visit to the practice to see how I was doing.
9. I was satisfied that my treatment was well organised.

**Possible answers:**

1 = never, 2 = rarely, 3 = sometimes, 4 = often, 5 = always

The indicator self-assessed quality of care was examined within the framework of the diabetes surveillance as part of the study Disease Knowledge and Information Needs – Diabetes mellitus (2017) conducted by the RKI [12]. In this nationwide health study, adults from the German-speaking resident population in Germany were assigned to two survey sections (representative sample or diabetes sample) using an established procedure. Data was obtained based on a telephone interview, which means that information from the study is self-reported. A detailed description of the study and the instruments used has been published elsewhere [12, 13].

The study enrolled 1,396 people with diagnosed diabetes in the past twelve months. People under 45 years of age were excluded from the analyses due to a small number of cases, as were those without complete information on self-assessed quality of care. The study population then comprised 1,254 participants (597 women, 657 men).

Data for the self-assessed quality of care indicator was collected using a German version of the Patient Assessment of Chronic Illness Care – DAWN short form (PACIC-DSF), which was adapted for diabetes [14]. The instrument comprises nine single questions, eight of which relate to central aspects of patient-oriented care such as patient’s wishes and goals in the treatment process and the impact of treatment on their daily life. The last question gathers data on satisfaction with the organisation of treatment overall (Info box). The questions relate to experiences made in the past twelve months and each could be answered with one of five possible answers. The sum of the numerical answer categories from the nine single questions divided by nine forms the PACIC-DSF score. The results are placed on a scale of 1 to 5, with higher values indicating better self-assessed quality of care.

The mean PACIC-DSF score, together with the corresponding 95% confidence interval (95% CI), serves as a measure of the level of self-assessed quality of care in the past twelve months. The results were calculated for the entire group and stratified by sex, age group, education level, population size of residential area and region. Differences with p values <0.05 were considered statistically significant.

A weighting factor was used to correct deviations from the underlying reference population caused by different participation or selection probabilities. This adapted the study sample to the population structure of the reference population (December 31, 2016) in terms of the distribution of sex, age and level of education. The distribution structure of people with diagnosed diabetes from the RKI’s German Health Update 2012 (GEDA 2012) was used for adjustment, since the data from the population statistics of the Federal Statistical Office do not facilitate conclusions to be drawn about the German-speaking population aged 18 years or above who are diagnosed with diabetes.

## Results and discussion

**Figure 1:**
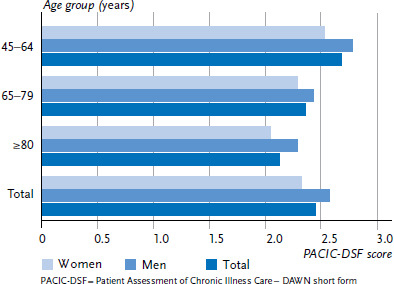
Mean PACIC-DSF score as a measure of the self-assessed quality of care in people aged 45 years or above and with diagnosed diabetes, both in the past twelve months, by sex and age (n=597 women, n=657 men) Source: Disease Knowledge and Information Needs – Diabetes mellitus (2017)

The mean self-assessed PACIC-DSF score (scale from 1 to 5) for quality of care in people with diagnosed diabetes in the past twelve months in the survey year 2017 was 2.45 (Figure 1). The mean value was 2.33 among women, significantly higher than that among men (2.58), which means that women provided a significantly poorer rating of the quality of the care they received than men. Figure 1 also demonstrates that significantly poorer self-assessments of care are associated with increasing age: the mean PACIC-DSF score in the age group 45 to 64 years is 2.68 compared to 2.13 among people aged 80 years or above. These patterns can be observed in both sexes.

In contrast, only few differences were identified in self-assessed quality of care by education level (Table 1). The mean values of the PACIC-DSF scores for the low, medium and high education group are all similar at 2.42, 2.49 and 2.43, respectively.

A poorer rating of quality of care was also associated with increasing population size of the residential area. The mean PACIC-DSF score is 2.62 for respondents living in a rural area or small town, but drops significantly to 2.33 for those living in a large city. A significant difference in self-assessed quality of care can also be seen between women and men by size of residential area. This pattern is observed among both women and men and is still present even if age has been taken into account (linear regression, data not shown).

The regional distribution of the PACIC-DSF score demonstrates a better self-assessment of quality of care in the central eastern region (Saxony, Saxony-Anhalt and Thuringia) with a mean score of 2.59 compared to 2.34 in the north-eastern region (Berlin, Brandenburg and Mecklenburg-Western Pomerania) and 2.37 in the central western region (Hesse, North Rhine-Westphalia, Rhineland-Palatinate and Saarland). The PACIC-DSF scores for the north-west (Bremen, Hamburg, Lower Saxony and Schleswig-Holstein) and south (Baden-Württemberg and Bavaria) are in between these figures (2.53 and 2.49 respectively, data not shown).

The results of this population-based study show that people with a diagnosed diabetes in the past twelve months assess the quality of the care they receive as moderate. Previous studies examining this indicator carried out in Germany have been based on clinical and regional study populations [15–18]; they found a similar or slightly better rating of quality of care than in the present study. Differences in the study design and the version of the PACIC questionnaire used mean that these results are only comparable to a limited extent. The values for quality of care determined by this study were calculated using a complex score derived from the results gained from nine single questions; the scores, therefore, were strongly influenced by the results from the questions about patient involvement in the treatment process. As such, they are not indicative of a general dissatisfaction with medical care, but rather illustrate an inadequate achievement of targets with regard to the patient-centred design of health care processes. In line with patient-centred health care provision to people with chronic illnesses, the PACIC questionnaire focuses on the questions that highlight the views of the people affected in treatment planning and communication with their doctors [17]. It is crucial that people with diabetes are asked how they are coping, how their illness affects their everyday life, how well they react to their medication, and what kind of supports they might need for self-management and towards achieving their treatment goals. Participation in training courses plays an important role here.

Until now, there is hardly any information available on self-assessed quality of care that could be aligned with sociodemographic factors. The sex-difference in self-assessed quality of care could be because women tend to find fault with care issues and expect more consideration from the treatment team. Various other instruments that have studied subjective perceptions have often shown that women tend to provide lower ratings than men. This has been the case with depressive symptoms [19] and self-assessed health [20] and variation in response behaviours may contribute towards this difference. In addition, it is also possible that a greater need for care and higher levels of psychosocial stress due to increasing health problems contribute to the lower ratings of quality of care that are generally provided by older people compared to younger people.

In summary, the PACIC-DSF score shows that people with diabetes in Germany tend to view the quality of their care as moderate. These results send a clear signal that improvements are needed in medical care provision, particularly in terms of a stronger focus on the needs of patients with diabetes, for example, in implementing treatment plans and treatment goals in their everyday life. The identification of population groups who assess their quality of care as poor highlights areas in which measures need to be put in place to improve the health care provided to people with diabetes. There is a great need for health services research in this area.

## Key statements

- People with diabetes in Germany assess the quality of their care as moderate.
- Women with diabetes provide a lower rating of their care than men with diabetes.
- Older people with diabetes tend to assess their care as poorer than younger people.
- There are slight regional but no educational differences in self-assessed quality of care.
- People with diabetes assess the quality of their care as poorer with increasing size of the population in their residential area.

## Figures and Tables

**Table 1:** Mean PACIC-DSF score as a measure of self-assessed quality of care among people aged 45 years or above and with diagnosed diabetes, both in the past twelve months, by education level, size of residential area and sex (n=597 women, n=657 men) Source: Disease Knowledge and Information Needs – Diabetes mellitus (2017)

Education level	PACIC-DSF score		Size of residential area	PACIC-DSF score	
	Mean	(95% CI)		Mean	(95% CI)
**Women**			**Women**		
Low education group	2.33	(2.18–2.48)	Rural/small town	2.55	(2.38–2.73)
Medium education group	2.30	(2.18–2.43)	Middle-sized town	2.28	(2.07–2.49)
High education group	2.39	(2.16–2.62)	Metropolitan area	2.27	(2.08–2.47)
**Men**			**Men**		
Low education group	2.53	(2.37–2.70)	Rural/small town	2.68	(2.53–2.82)
Medium education group	2.67	(2.54–2.80)	Middle-sized town	2.55	(2.33–2.76)
High education group	2.45	(2.31–2.59)	Metropolitan area	2.38	(2.25–2.52)
**Total**			**Total**		
Low education group	2.42	(2.31–2.53)	Rural/small town	2.62	(2.51–2.73)
Medium education group	2.49	(2.40–2.59)	Middle-sized town	2.42	(2.27–2.57)
High education group	2.43	(2.31–2.55)	Metropolitan area	2.33	(2.22–2.45)

PACIC-DSF = Patient Assessment of Chronic Illness Care – DAWN short form, CI = confidence interval
